# *In Vivo* and *Ex Vivo* Evaluation of 1,3-Thiazolidine-2,4-Dione Derivatives as Euglycemic Agents

**DOI:** 10.1155/2021/5100531

**Published:** 2021-12-31

**Authors:** Diana Alemán-González-Duhart, Samuel Álvarez-Almazán, Miguel Valdes, Feliciano Tamay-Cach, Jessica Elena Mendieta-Wejebe

**Affiliations:** ^1^Laboratorio de Biofísica y Biocatálisis, Sección de Estudios de Posgrado e Investigación, Escuela Superior de Medicina, Instituto Politécnico Nacional, Plan de San Luis y Salvador Díaz Mirón s/n, Casco de Santo Tomás, Miguel Hidalgo, 11340 Ciudad de México, Mexico; ^2^Laboratorio de Investigación en Bioquímica Aplicada, Sección de Estudios de Posgrado e Investigación y Departamento de Formación Básica Disciplinaria, Escuela Superior de Medicina, Instituto Politécnico Nacional, Plan de San Luis y Salvador Díaz Mirón s/n, Casco de Santo Tomás, Miguel Hidalgo, 11340 Ciudad de México, Mexico; ^3^Departamento de Formación Básica Interdisciplinaria, Centro Interdisciplinario de Ciencias de la Salud-Unidad Santo Tomás, Instituto Politécnico Nacional, Av. de los Maestros s/n, Casco de Santo Tomás, Miguel Hidalgo, 11340 Ciudad de México, Mexico; ^4^Laboratorio 4 de Biotecnología, Facultad de Estudios Superiores Cuautitlán, Campo 1, Universidad Nacional Autónoma de México, Avenida 1° de Mayo s/n, Santa María las Torres, Cuautitlán Izcalli, 54740 Estado de México, Mexico

## Abstract

Thiazolidinediones (TZDs), used to treat type 2 diabetes mellitus, act as full agonists of the peroxisome proliferator-activated receptor gamma. Unfortunately, they produce adverse effects, including weight gain, hepatic toxicity, and heart failure. Our group previously reported the design, synthesis, in silico evaluation, and acute oral toxicity test of two TZD derivatives, compounds 40 (C40) and 81 (C81), characterized as category 5 and 4, respectively, under the Globally Harmonized System. The aim of this study was to determine whether C40, C81, and a new compound, C4, act as euglycemic and antioxidant agents in male Wistar rats with streptozotocin-induced diabetes. The animals were randomly divided into six groups (*n* = 7): the control, those with diabetes and untreated, and those with diabetes and treated with pioglitazone, C40, C81, or C4 (daily for 21 days). At the end of the experiment, tissue samples were collected to quantify the level of glucose, insulin, triglycerides, total cholesterol, and liver enzymes, as well as enzymatic and nonenzymatic antioxidant activity. C4, without a hypoglycemic effect, displayed the best antioxidant activity. Whereas C81 could only attenuate the elevated level of blood glucose, C40 generated euglycemia by the end of the treatment. All compounds produced a significant decrease in triglycerides.

## 1. Introduction

Type 2 diabetes mellitus (T2DM) is a chronic metabolic disease characterized by hyperglycemia. It is manifested as an elevated level of blood glucose (>126 mg/dL) and glycosylated hemoglobin (>6.5%), peripheral insulin resistance, and an excessive accumulation of triglycerides and derivatives of fatty acids in skeletal muscle and other tissues. These conditions can give rise to micro- and macrovascular complications [1–3].

Chronic hyperglycemia fosters metabolic alterations through the deregulation of signal transduction. The resulting modification in the expression of a variety of genes leads to tissue damage and a proinflammatory environment, which are directly responsible for the development of many complications associated with T2DM [4, 5].

The treatment of T2DM has focused on lowering blood glucose by increasing the secretion of insulin or decreasing resistance to this hormone in peripheral tissues. Thiazolidinediones (TZDs), commonly used for such treatment, act as full agonists of the peroxisome proliferator-activated receptor gamma (PPAR*γ*) [6–8], which is involved in the pathophysiology of various diseases apart from T2DM and obesity, including dyslipidemia, atherosclerosis, neoplasia and tumors, inflammatory disorders, and neurodegenerative diseases [9–11].

TZDs are constituted by a hydrophilic head, an aromatic body, and a cyclic tail. Since commercially available TZDs contain a stereogenic center at carbon 5 of the hydrophilic head, they are susceptible to the formation of a racemic mixture through physiological processes. Only the (S) enantiomer of the mixture binds to the receptor, leaving approximately 50% of the drug without activity. This characteristic lends itself to adverse effects [12–15], among which are fluid retention, weight gain, hepatic toxicity, plasma volume expansion, hemodilution, edema, and heart failure [6, 16, 17].

Several groups have used the TZD pharmacophore to design, synthesize, and evaluate new molecules for the treatment of different ailments, achieving an improvement in hypoglycemic activity and a decrease in adverse effects [18–20]. However, satisfactory results have not yet been obtained. The best *in vivo* euglycemic activity has been found with molecules bearing halide versus hydroxyl group substituents on the tail. Effective halide substituents are mainly located in the *ortho* and *meta* positions. Whereas the tail has been successfully modified, the other two portions of the new molecules are the same as those existing in commercially available drugs [21].

Our group has reported the design and synthesis of two TZD derivatives, denominated compounds 40 (C40) and 81 (C81) [22]. C40 consists of the polar head, 1,3-thiazolidine-2,4-dione, and salicylaldehyde, while C81 contains the polar head and 2-fluoro-4-chlorobenzaldehyde. Both compounds interact with PPAR*γ* in a way similar to other known full agonists, thus suggesting a similar mechanism of action. C40 and C81 do not generate any evident toxic effect, a finding derived from the application of protocol 425 of the Organization for Economic Cooperation and Development (OECD) [22]. They have been characterized as categories 5 and 4, respectively, under the Globally Harmonized System.

The aim of the present study was to explore the possible euglycemic and antioxidant activity of C40, C81, and a newly synthesized TZD derivative, designated as compound 4 (C4). These compounds have an adequate profile for the effective treatment of T2DM without producing the classic toxicity exhibited by other drugs in the TZD family, such as pioglitazone, troglitazone, and rosiglitazone.

## 2. Materials and Methods

### 2.1. Chemicals

Urea, 2,4-thiazolidinedione, streptozotocin, pioglitazone hydrochloride, cinnamaldehyde, sodium citrate, citric acid anhydrous, sodium chloride, glacial acetic acid, dimethyl sulfoxide, ascorbic acid, D-glucose, sodium pentobarbital, and ethylenediaminetetraacetic acid were purchased from Sigma Chemical Co. (St. Louis, MO, USA).

### 2.2. Animals

Forty-two healthy male albino Wistar rats weighing 170 ± 20 g (UPEAL Bioterium, UAM-Xochimilco, Mexico City, Mexico) were housed 3-4 animals per cage for 42 days (6 weeks). They were kept on a 12/12 h light/dark cycle in a well-ventilated room at 22 ± 3°C with 30-35% relative humidity and given a conventional rodent laboratory diet (Rat Chow 5012) and drinking water *ad libitum* throughout the study. The experiments were conducted in accordance with the guidelines for animal research from the National Institutes of Health and the Mexican official norm (NOM-062-ZOO-1999) [21, 23–25]. The protocol was approved by the Committee for the Care and Use of Laboratory Animals (CICUAL-10/21-06-2017) at the Escuela Superior de Medicina, Instituto Politécnico Nacional, Mexico City, Mexico.

### 2.3. Chemical Synthesis

**Figure 1:**
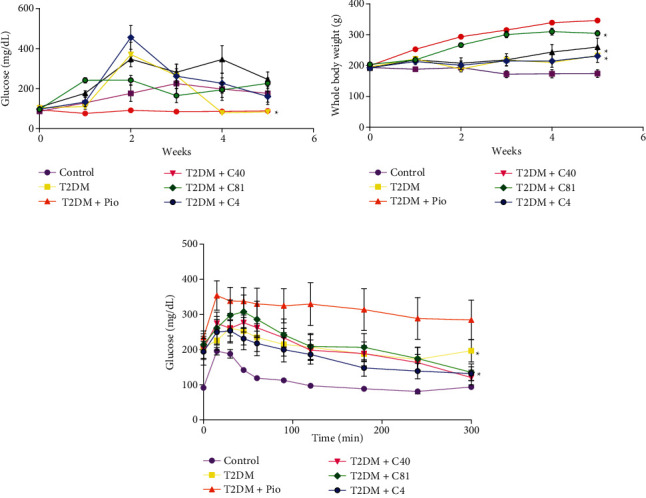
(a) The fasting blood glucose level was evaluated in all groups (*n* = 7). ^∗^*p* ≤ 0.05 vs. T2DM. (b) Body weight of the animals subjected to the different treatments (*n* = 7). ^∗^*p* ≤ 0.05 vs. T2DM. (c) The glucose tolerance test from 0 to 300 min. Compared to the untreated diabetic rats, the animals treated with derivatives C40, C81, and C4 displayed a lower level of blood glucose at the end of the experiment (*n* = 7). ^∗^*p* ≤ 0.05 vs. T2DM+Pio (diabetic rats treated with pioglitazone). T2DM, untreated diabetic rats.

The reaction sequence employed for the synthesis of the proposed compounds C4, C40, and C81 was based on a Knoevenagel condensation, using equimolar concentrations and a catalytic amount of urea at 10 mol % in a solvent-free environment. 2,4-Thiazolidinedione can undergo a Knoevenagel condensation with a variety of substituted aldehydes to produce 5-arylidene-2,4-thiazolidinediones (Figure 1, Supplementary material (available here)). All the synthesized compounds were characterized by spectroscopic methods such as infrared (IR), ^1^H and ^13^C nuclear magnetic resonance (NMR), and mass spectrometry (MS) [22].

### 2.4. In Vivo Evaluation of Compounds C40, C81, and C4

The rats were allowed 1 week of acclimation to lab conditions before carrying out the 5-week experiment. The beginning of the experiment was considered week 0 (W0), at which time each rat was weighed, and blood samples were taken from the tail vein for the first measurement of the blood glucose level. T2DM was then induced by a single intraperitoneal (i.p.) injection of streptozotocin (STZ) (Sigma Chemical Co., St Louis, MO, USA) in each rat of five groups, a procedure omitted for the healthy nondiabetic control animals. STZ was dissolved in 0.01 M sodium citrate buffer (pH 4.5) and administered in a single dose of 45 mg/kg body weight. Seven days later, denominated week 1 (W1), the tail vein blood glucose level was measured with a glucometer (Accu-Check Active, Roche, Germany) and reactive strips (Accu-Check Active Glucose test strips, Roche, Germany). All rats with blood glucose levels over ≥126 mg/dL were considered diabetic.

The rats were randomly divided into six groups (*n* = 7): the control (basal), those with diabetes and untreated (T2DM), and those with diabetes and treated with pioglitazone (30 mg/kg/day, as a reference), C40 (18 mg/kg/day), C81 (21 mg/kg/day), or C4 (19 mg/kg/day). Treatments were administered daily at the same time of day in a volume of 1 mL/100 g body weight per day via gavage from the beginning of week 2 (W2) to the end of week 4 (W4), constituting 21 days. All doses were prepared in an equimolar relation to the pioglitazone dose. At the end of the treatment, all animals were deeply anesthetized with 72 mg/kg sodium pentobarbital to take blood and tissue samples. Whole blood was collected by cardiac puncture (using ethylenediaminetetraacetic acid (EDTA) as an anticoagulant) and centrifuged at 2000 rpm for 15 min to obtain erythrocytes and plasma, which were used to determine glucose, insulin, antioxidant, and liver enzymatic activities. The liver was removed and washed with phosphate-buffered saline (PBS) to assess nonenzymatic activity [23].

### 2.5. The Glucose Tolerance Curve

Glucose tolerance was examined in all groups by i.p. injection of D-glucose (2 g/kg, 20% *w*/*v* saline) after 6 h of fasting. The blood glucose level was measured as aforementioned and monitored for 120 min [26, 27].

### 2.6. Ex Vivo Evaluation of C40, C81, and C4

#### 2.6.1. Plasma Glucose and Insulin

The plasma glucose concentration was quantified by means of the glucose oxidase method [26–29] and the plasma insulin level by an enzymatic immunoassay, in both cases with a commercially available kit (glucose with Gluc-Pap, Randox, No. Cat. GL2614; insulin with Kit Spibio, Randox, No. Cat. A05105) [26, 28, 30].

#### 2.6.2. Total Cholesterol and Triglycerides

Total cholesterol and triglyceride levels were determined with an enzymatic colorimetric test from commercially available kits (CHOL, Randox, CH200; GPO-PAP, Randox, No. Cat. TR210), in accordance with the manufacturer's instructions [26, 31].

#### 2.6.3. Enzymatic Antioxidant Activity

Superoxide dismutase (SOD) activity was evaluated by an indirect method using a commercial kit (RANSOD, Randox, No. Cat. SD125), which allows for the differential quantification of mitochondrial and cytosolic SOD activity by inhibition of the latter. SOD activity is expressed in activity units, one unit being the amount of enzyme capable of inhibiting 50% of cytochrome c reduction in a system coupled with xanthine oxidase [26, 32, 33]. Catalase (CAT) activity was examined in plasma with a commercial kit (Cayman Chemical, USA), following the manufacturer's instructions [26, 34].

#### 2.6.4. Nonenzymatic Antioxidant Activity

A portion of frozen liver sample (0.1 g) was homogenized in PBS (at pH 8 for reduced glutathione (GSH) and pH 7.4 for malondialdehyde (MDA)) and then centrifuged at 6000 rpm for 30 min at 4°C. Clear supernatants were separated and employed for the assessment of GSH and MDA. Since the reduced form of glutathione comprises the bulk of the cellular nonprotein sulfhydryl group, this technique is based on the development of a stable yellow solution when 5,5′-ditiobis-2-nitrobenzoic acid (DTNB) is added to a sulfhydryl compound. Absorbance was measured at 412 nm, and the GSH value was estimated from a standard GSH curve [35, 36].

The MDA level was established by using the thiobarbituric acid (TBA) assay, which is based on the ability of MDA to react with TBA in an acidic medium at 95°C for 1 h. A pink thiobarbituric acid reactive substance (TBARS) is formed and quantified at 532 nm. The value of MDA is then taken from a standard 1,1,3,3-tetramethoxypropane 99% (TMP) curve for each sample [37].

#### 2.6.5. Hepatic Function

To evaluate hepatic damage, the activity of aspartate aminotransferase (AST), alanine aminotransferase (ALT), and alkaline phosphatase (ALP) was determined in plasma by enzymatic methods with commercial kits (No. Cat. AS1267, AL1268, and AP307, Randox, USA), according to the manufacturer's instructions [26, 38].

### 2.7. Statistical Analysis

Statistical analysis was performed on SigmaStat 3.5, and all data were expressed as the mean ± standard deviation. Comparisons between groups were made with one-way ANOVA. A level of probability of *p* ≤ 0.05 was set as statistically significant. Graphs were constructed on GraphPad Prism 5.0.

## 3. Results

### 3.1. Fasting Blood Glucose Level and Body Weight

The level of blood glucose ranged from 76.16 to 94.16 mg/dL (W1-W4) in the control group (basal) and from 129.42 to 225.85 mg/dL (considered hyperglycemia) in the untreated diabetic group. The final glucose level, at the end of W4, was 246.14 ± 36.98 mg/dL for pioglitazone-treated rats and 226.85 ± 26.81 mg/dL for C4-treated animals, indicating that these compounds did not decrease the level of blood glucose (Figure 1(a)).

In contrast, the final blood glucose level was slightly lower in C81-treated rats (gradually declining from 456.37 ± 59.39 to 160.85 ± 27.41 mg/dL) versus the untreated diabetic group. Even though the final value still represents hyperglycemia, C81 was able to reduce glycemia by ~300 mg/dL. On the other hand, the C40 treatment exhibited the desired effect of decreasing the blood glucose level as of W3. This parameter diminished in the C40-treated animals from 371.0 ± 61.72 mg/dL after the administration of STZ (W1) to 84.0 ± 3.82 mg/dL by the end of the experiment (W4) (Figure 1(a)). Hence, the final value was significantly lower than that of the untreated diabetic group.

Regarding body weight, all groups started at 200.0 ± 10.0 g. The control group (basal) displayed a normal time-dependent increase in body weight, with an overall increment at the end of W4 of 146.49 g. As expected, the untreated animals with STZ-induced diabetes exhibited caquexia, indicated by a decline in their original weight of 193.81 ± 3.30 g to a final value of 174.14 ± 12.48 g. The four compounds tested presently were all adipogenic agents. The weight gain was 67.86 g in the pioglitazone-treated group (from 192.14 ± 1.03 g to 260.0 ± 28.57 g), 36.8 g in the C40-treated group, and 37.85 g in the C81-treated group. The weight gained in the latter two groups represents about 50% of that found with the pioglitazone treatment. The weight gain in the C4-treated group was 100.82 g, almost twice the amount shown by the pioglitazone-treated animals (Figure 1(b)).

### 3.2. Glucose Tolerance Test

In the glucose tolerance test (Figure 1(c)), the area under the curve was 91.5 ± 5.10 mg/dL at time 0 in the control group (basal). After administering 1.5 g/kg of glucose, the concentration rose significantly to 195.66 ± 10.71 mg/dL by minute 15. The level began to fall at minute 30 and reached a value of 118.83 ± 5.09 mg/dL, considered as euglycemia, by minute 60. From this moment on, the curve of the control group remained in a status of euglycemia until the end of the assay at minute 120.

All five diabetic groups (untreated or with one of the four treatments) had over 200 mg/dL of blood glucose at minute 0. After administering 1.5 g/kg of glucose, the concentration showed an increase at minute 15 and began to descend by minute 45. The C40 treatment resulted in a value of 120.57 ± 20.72 mg/dL of glucose, the C81 treatment in 135.42 ± 24.11 mg/dL, and the C4 treatment in 131.71 ± 19.40 mg/dL at minute 120, demonstrating that C40 is the most effective of these possible postprandial hypoglycemic agents. Indeed, it was capable of generating postprandial euglycemia by the end of the 3-week treatment (Figure 1(c)).

### 3.3. Ex Vivo Assays

#### 3.3.1. Plasma Glucose and Insulin

**Figure 2:**
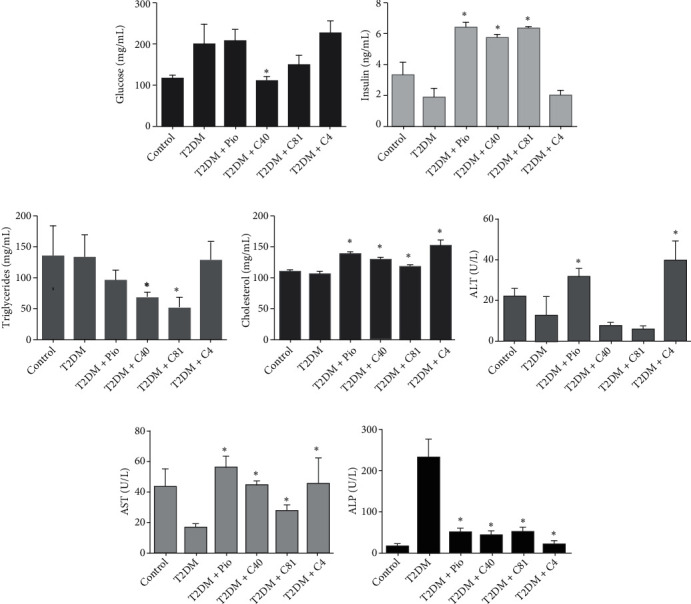
Metabolic parameters of the different groups (*n* = 7): (a) glucose (mg/dL), (b) insulin (ng/mL), (c) triglycerides (mg/dL), (d) cholesterol (mg/dL), (e) ALT (U/L), (f) AST (U/L), and (g) ALP (U/L). ^∗^*p* ≤ 0.01 vs. the untreated diabetic group (T2DM). Pio: pioglitazone.

A normal blood glucose value of 115.48 ± 8.54 mg/dL was found in the control group (basal) and a significantly higher level of 200.78 ± 28.70 mg/dL in the untreated diabetic group by the end of the 5-week experiment. The blood glucose concentration was still in a hyperglycemia status (at 208.81 ± 28.70 mg/dL) after the 3-week treatment with pioglitazone, and even higher (228.92 ± 28.34 mg/dL) with C4. Although C81 produced a significant reduction of 150.56 ± 23.84 mg/dL by the end of the 3-week treatment, the resulting level does not indicate euglycemia. On the other hand, the C40 treatment effectively decreased plasma glucose to 112.46 ± 9.43 mg/dL, reaching a euglycemic level by the end of the 3-week treatment (Figure 2(a)).

The concentration of insulin in the control group (basal) was 3.15 ± 0.72 ng/mL. The values of 1.87 ± 0.57 ng/mL for the untreated diabetic rats and 2.00 ± 0.37 ng/mL for the C4-treated animals were slightly (but not significantly) lower. Compared to the untreated diabetic rats, there was a significant increase in the insulin concentration for the animals receiving either of the other three treatments: 6.42 ± 0.30 ng/mL for pioglitazone, 5.77 ± 0.20 ng/mL for C40, and 6.37 ± 0.01 ng/mL for C81 (Figure 2(b)).

#### 3.3.2. Total Cholesterol and Triglycerides

The level of triglycerides in the control group (basal) was 138.81 ± 48.87 mg/dL, and that of three other groups was not significantly different: 133.12 ± 37.89 mg/dL for the untreated diabetic group, 96.78 ± 16.41 mg/dL for the pioglitazone group, and 129.88 ± 29.90 mg/dL for the C4 group. Compared to the untreated diabetic rats, the level of triglycerides was significantly lower for the C40- and C81-treated animals, being 68.59 ± 8.01 mg/dL and 52.14 ± 16.78 mg/dL, respectively (Figure 2(c)).

The level of total cholesterol was not significantly different between the control and untreated diabetic groups, being 110.79 ± 2.67 mg/dL and 107.23 ± 3.95 mg/dL, respectively. Compared to the untreated diabetic group, the level of total cholesterol increased significantly with all the treatments, being 138.69 ± 4.41 mg/dL for pioglitazone, 130.21 ± 3.26 mg/dL for C40, 118.65 ± 3.65 mg/dL for C81, and 154.26 ± 6.92 mg/dL for C4 (Figure 2(d)).

The plasma concentration of ALT was not significantly different between the control and untreated diabetic groups, being 21.79 ± 4.29 U/L and 12.21 ± 9.27 U/L, respectively. Compared to the untreated diabetic group (12.21 ± 9.27 U/L), nonsignificantly lower values were found for the C40- and C81-treated rats, being 7.27 ± 1.66 U/L and 5.44 ± 1.68 U/L, respectively. Contrarily, a significantly higher level was detected in the pioglitazone- and C4-treated animals, being 31.57 ± 4.20 U/L and 39.32 ± 9.96 U/L, respectively (Figure 2(e)). Considering the fluctuations in ALT activity between groups, all levels remained within normal parameters (<45 U/L for human beings or rats).

Plasma AST activity for the control group (basal) was 42.35 ± 12.55 U/L. The level in the untreated diabetic group was 16.22 ± 2.93 U/L, representing a significant decrease (Figure 2(f)). Compared to the latter value, all the treatments significantly enhanced AST activity, reaching 55.60 ± 7.80 U/L with pioglitazone, 44.14 ± 2.40 U/L with C40, 27.18 ± 3.92 U/L with C81, and 44.98 ± 17.37 U/L with C4. An increase in AST does not produce any clinical symptoms, but a value below 20 U/L may be an indicator of kidney damage, as observed in the untreated diabetic group.

ALP activity was 16.75 ± 6.36 U/L in the control group (basal) and slightly (nonsignificantly) higher in the treated groups, being 52.44 ± 9.52 U/L with pioglitazone, 42.97 ± 11.54 U/L with C40, 49.94 ± 14.25 U/L with C81, and 21.42 ± 7.94 U/L with C4. Contrarily, significantly greater activity was found for the untreated diabetic group, reaching 234.65 ± 44.52 U/L (Figure 2(g)).

#### 3.3.3. Enzymatic and Nonenzymatic Antioxidant Activity

**Figure 3:**
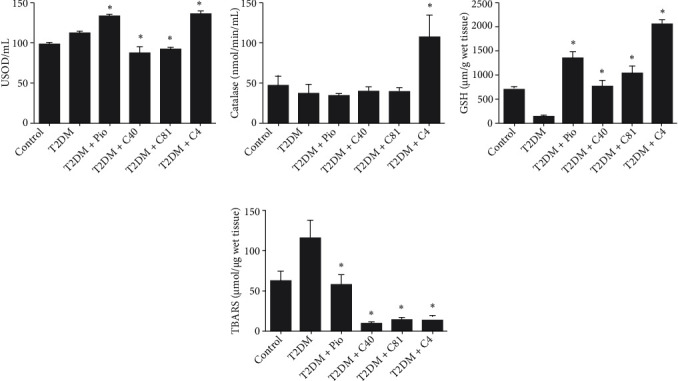
Enzymatic and nonenzymatic antioxidant activity in the different groups (*n* = 7): (a) SOD (U/mL), (b) CAT (nmol/min/mL), (c) GSH (*μ*M/g of wet tissue), and (d) TBARS (*μ*mol/*μ*g of wet tissue). ^∗^*p* ≤ 0.01 vs. T2DM (the untreated diabetic rats). Pio: pioglitazone.

There was no significant difference between the SOD activity of 99.06 ± 0.49 U/L in the whole blood of the control group (basal) and the corresponding level detected in the C40- and C81-treated groups, being 88.09 ± 8.72 U/L and 98.48 ± 1.95 U/L, respectively. These values were significantly lower than that found in the untreated diabetic rats and the 133.66 ± 1.99 and 136.34 ± 2.87 U/L observed in the pioglitazone- and C4-treated animals, respectively (Figure 3(a)).

Plasma CAT activity in the control group (basal) was 46.61 ± 12.51 nmol/min/mL, not significantly different from the 37.05 ± 11.10 nmol/min/mL of the untreated diabetic rats, or the values exhibited by the pioglitazone-, C40-, and C81-treated animals, being 33.07 ± 3.77, 39.36 ± 5.65, and 39.80 ± 4.44 nmol/min/mL, respectively. However, a significantly greater level of 106.78 ± 28.12 nmol/min/mL was displayed by the C4-treated animals, reaffirming the possibility of an antioxidant potential for this compound (Figure 3(b)).

The concentration of GSH in hepatic tissue was 700.95 ± 43.09 *μ*M/g for the control rats (basal) and a significantly lower 116.91 ± 27.48 *μ*M/g for the untreated diabetic animals. There was no significant difference between the GSH level of the control and treatment groups, evidenced by the GSH level of 1337.28 ± 141.81 *μ*M/g for pioglitazone, 750.11 ± 118.01 *μ*M/g for C40, 1016.88 ± 153.08 *μ*M/g for C81, and 2053.25 ± 77.60 *μ*M/g for C4 (Figure 3(c)).

Regarding TBARS, a concentration of 63.58 ± 16.06 *μ*mol/*μ*g was found in the hepatic tissue of the control group (basal) and a significantly higher level of 116.16 ± 22.23 *μ*mol/*μ*g was detected in the untreated diabetic rats. Compared to the latter group, a significantly lower value was observed for the animals subjected to each of the four treatments: 57.30 ± 13.58 *μ*mol/*μ*g for pioglitazone, 9.39 ± 1.29 *μ*mol/*μ*g for C40, 14.06 ± 3.85 *μ*mol/*μ*g for C81, and 13.96 ± 5.62 *μ*mol/*μ*g for C4 (Figure 3(d)).

## 4. Discussion

T2DM causes chronic and progressive damage, leading to deteriorating health and high medical costs. Due to the importance of finding new therapeutic alternatives capable of reducing or controlling the effects of this disease, hypoglycemic activity was presently assessed for three TZD derivatives: C40, C81, and C4.

The T2DM model adopted for the current contribution was adequate for examining the euglycemic and antioxidant effects of the tested compounds, as demonstrated by the level of insulin. The limitation of the model is the exclusion of other metabolic parameters (e.g., hyperinsulinemia and hypercholesterolemia), a shortcoming that will be taken into account when choosing a model for future studies.

According to the ex vivo parameters, the C40 treatment effectively decreased the blood glucose level in diabetic rats to a euglycemic level, which may be due to several factors. Firstly, C40 possibly stimulates the transcription of proteins involved in carrying out and regulating carbohydrate homeostasis, such as glucose transporters 1 (GLUT1) and 4 (GLUT4). These two isoforms are found in adipose tissue, liver, and skeletal muscle, thus facilitating the provision of insulin-mediated glucose to peripheral tissues. Secondly, TZDs and their derivatives are known to inhibit gluconeogenesis, another route that perhaps participates in the euglycemic effects of C40 [39, 40]. Thirdly, TZDs can inhibit the signaling pathway of vascular endothelial growth factor (VEGF) and the synthesis of proinflammatory cytokines. As a result, peripheral insulin sensitivity is enhanced, leading to an increased consumption of glucose in skeletal muscle and heart tissue and a consequent decrease in the level of blood glucose [7].

Considering the hypothesis that C40, C81, and C4, being TZD derivatives, bind to PPAR*γ* to normalize blood glucose, the positive results with C40 were plausibly favored by the presence of electron-donating substituents on the aromatic ring of this compound. The presence of an electron-withdrawing substituent, such as halogens in C81, could have also helped to lower blood glucose, but to a lesser extent. In contrast, the lack of a decrease in the level of blood glucose with the C4 treatment might be associated with the absence of substituents on the aromatic ring and/or the presence of more than one carbon atom as a spacer between the aromatic and TZD rings [21]. These structural differences likely played a role in the distinct metabolic and antioxidant effects produced by the treatments.

TZDs activate AMP-activated protein kinase (AMPK) in the liver, which directly improves hepatic insulin sensitivity, facilitates the oxidation of fatty acids, and diminishes the synthesis of fatty acids and triglycerides [41–44]. Treatment with pioglitazone, C40, C81, and C4 caused a reduction in the triglyceride levels (compared to the untreated diabetic group), an effect previously described for full PPAR*γ* agonists as well as dual *α*/*γ* agonists [19, 30, 45–48].

DePaoli et al. mentioned that pioglitazone treatment tends to diminish the level of low-density lipoprotein (LDL), very low-density lipoprotein (VLDL), and total cholesterol [46], which is corroborated in the current study by a decrease in the levels of total cholesterol. This effect has been explained by Soccio et al. as a possible partial agonism of PPAR*α* by TZDs [49]. Additionally, the mechanism of action of these PPAR*γ* agonists is known to generate a lower level of plasma triglycerides, an increase in high-density lipoproteins (HDL), and a decline in LDL and VLDL. In future research, therefore, a change to a high-fat diet is suggested for animals treated with C40 or C81, along with a separate quantification of each of the lipoproteins [9, 11].

Antioxidant enzyme activity was not significantly different between the untreated diabetic rats and those treated with C40 or C81. Contrarily, the C4 treatment afforded significantly greater CAT and SOD activity, in agreement with the findings of Assaei et al. [24]. In this sense, it is known that the Cu/Zn-SOD gene is closely related to the nuclear factor kappa B (NF-*κ*B). The latter redox-sensitive transcription factor acts as a regulator of genes and plays a role in cell injury. During NF-*κ*B activation, oxidation-reduction can be caused by hydrogen peroxide (H_2_O_2_), generated in the reaction catalyzed by Cu/Zn-SOD on the endosomal surface. Such oxidation-reduction leads to greater Cu/Zn-SOD expression. Moreover, the increase in the dismutation rate of a superoxide anion radical results in the accumulation of H_2_O_2_. The amount of CAT is known to be controlled by the presence of the substrate [50].

On the other hand, the gene of these enzymes contains a PPAR binding domain (Refaat, [51]). Based on experimental evidence, PPAR*γ* agonists may exert their anti-inflammatory activity by diminishing the production of proinflammatory cytokines (e.g., TNF-*α*, IL-2, IL-6, and IL-8). This would improve the bioavailability of nitric oxide, which elicits the expression and activity of antioxidant enzymes (e.g., SOD) and suppresses the generation of the superoxide anion by NADPH oxidase [52, 53].

According to some reports, TZD derivatives and other groups of drugs can establish an intrinsic antioxidant activity (due to their structure) and also trigger the synthesis or activation of endogenous antioxidant molecules [54, 55]. A molecule capable of decreasing the amount of ROS can protect against cell damage and apoptosis [50]. Many researchers have suggested that the presence of conjugated double bonds throughout a molecule (as in the case of C40) can give intrinsic antioxidant properties through free radical scavenging [54, 56, 57].

A potentially important characteristic of C40 is the presence of nitrogen on the heteroatomic ring (as occurs with melatonin), functioning as a secondary amine that quenches the production of ˙OH. This proceeds by the chelation of copper (II) and/or iron (III) in the organism with a Fenton reaction [55]. Another suggested antioxidant activity of flavonoids is their capacity to donate a hydrogen atom or an electron from the hydroxyl group on the ring, followed by their stabilization by resonance [58]. Such activity may be shown by the amino group of the TZD acid ring.

Although halide substituents on the aromatic ring of glitazones favor hypoglycemic effectiveness, they appear to decrease the intrinsic antioxidant capacity of the molecule [21]. The existence of an electron donor, as in C40, increases the electron density of the aromatic ring, resulting in a higher electron density in the TZD acid ring that can cause an oxidation interaction with free radicals [59]. Hence, the C40-induced reduction in the levels of glucose might be related to the antioxidant properties of this compound.

The imbalance between oxidative stress and the antioxidant defense is a major factor in the negative effects of diabetes [60]. Oxidative stress has been correlated with glycemic variability. Several inducers of insulin resistance, including proinflammatory cytokines and oxidative stress, activate the expression of inducible nitric oxide synthase (iNOS), leading to the excessive NO production involved in the pathogenesis of T2DM when linked to insulin resistance and obesity [51]. During the development of T2DM, there are greater levels of the superoxide anion produced by the mitochondria and of cytochrome P450, xanthine oxidase, and NADPH oxidase.

On the other hand, the end products of glycosylation and/or the free radicals generated during the autoxidation of glucose can initiate the lipoperoxidation of lipoproteins related to the formation of MDA. An elevated MDA level is known to be an important marker of *in vivo* lipid peroxidation. A high concentration of lipoperoxidation products can lead to the formation of pores in the membrane and a hardening of this cell surface through the downregulation of unsaturated fatty acids. This in turn can influence the state of insulin receptors, bringing about a lower glucose consumption by cells [50].

According to Assaei et al., pioglitazone treatment can significantly decrease the amount of MDA as well as increase CAT activity. The current results corroborate this finding, demonstrating the same effect by the present TZD derivatives Assaei, [24]. In other studies with distinct experimental conditions, a similar behavior has been observed in relation to the levels of MDA, GSH, and the activity of the antioxidant enzymes SOD, CAT, and GPx [51, 61–65].

STZ-induced diabetes involves a prooxidant environment, manifested as a decline in the level of hepatic GSH and an elevated level of MDA. The latter, a result of lipid peroxidation, is generated by alterations in lipid metabolism that lead to an overproduction of peroxides and the inhibition of peroxidase activity [24]. These characteristics of the STZ model were herein confirmed by the data from the untreated diabetic group (T2DM). All the treatments given to the diabetic rats (pioglitazone, C40, C81, and C4) reversed the STZ-induced decrease in GSH and reduced the hepatic impairment caused by a higher level of MDA. The same outcome was previously described for TZD. Such regulation of oxidative stress markers by the present TZD derivatives is consistent with reports in the literature showing that this class of compounds has antioxidant and free radical scavenging properties [24, 51, 52, 66, 67].

The hypothetical potential hepatic toxicity of the test compounds was discarded based on the normal values found for ALT and AST (<40 U/L) [68]. Pioglitazone treatment lowers serum ALT and AST levels, which improves the condition of hepatic steatosis and inflammation caused by impaired glucose tolerance and/or insulin resistance [68–70]. Such an effect may be explained by the enhanced levels of adiponectin triggered by TZD treatment, leading to a greater flow of free fatty acids, a boost in fatty acid oxidation, and a lower level of inflammation [69, 71, 72].

ALP, considered a parameter of bone metabolism, together with procollagen type 1 N-terminal propeptide is widely used as a marker of bone formation [73]. Some studies in humans and animal models have examined bone markers following TZD treatment. Pioglitazone treatment is known to trigger a significant reduction in serum ALP, which has been suggested to indicate a decline in bone formation with no change in resorption [73, 74]. This previously reported decrease in serum ALP was corroborated presently for pioglitazone and the TZD derivatives (C40, C81, and C4).

## 5. Conclusion

In the current model of diabetic rats, the C40 treatment lowered blood glucose to a euglycemic level, evidenced by the *in vivo* and ex vivo evaluations. The administration of C81 also diminished blood glucose, but the effect was not sufficient to establish euglycemia. Although C4 did not lower blood glucose levels, it increased enzymatic and nonenzymatic antioxidant activity. All the treatments produced a significant decrease in triglycerides, which suggests their possible use to treat metabolic syndrome.

## Data Availability

The data set presented here in order to support the findings of this study is included within the article. Additional data analyzed is available in the supplementary material.

## Supplementary Materials

Supplementary Materials Synthesis, docking studies and acute toxicity test of compounds.
