# Supplementary material for: In Vivo and Ex Vivo Evaluation of 1,3-Thiazolidine-2,4-Dione Derivatives as Euglycemic Agents
Source: PPAR Res. 2021 Dec 31;2021:5100531. doi: 10.1155/2021/5100531 (PMC8741387; doi:10.1155/2021/5100531)
Supplement: Supplementary Materials — Synthesis, docking studies and acute toxicity test of compounds. [file 5100531.f1.docx]

**Synthesis, *docking* studies and acute toxicity test of compounds**

1. *Methodology*
   1. *Synthesis* *of compounds C4, C40, and C81*

C4, C40, and C81 were synthesized as previously reported [Alemán *et al*., 2017; Sahiba *et al*., 2020; Sahiba and Agarwal, 2020; Sahiba *et al*., Agarwal *et al*., 2014], with 1,3-thiazolidine-2,4-dione (1.173 g, 1 mmol; CAS: 2295-31-0 Sigma-Aldrich, Toluca, Estado de México, México) for the three compounds, and for C4 was used cinnamaldehyde (1.321 g, 1 mmol; CAS: 104-55-2 Sigma-Aldrich, Toluca, Estado de México, México), for C40 was used salicylaldehyde (1.221 g, 1 mmol; CAS: 90-02-8 Sigma-Aldrich, Toluca, Estado de México, México), and for C81 was used 3-chloro-2-fluorobenzaldehyde (1.585 g, 1 mmol; CAS: 85070-48-0 Sigma-Aldrich, Toluca, Estado de México, México). This solvent-free reaction was carried out at 120 °C for 2 h based on a Knoevenagel condensation, using equimolar concentrations and a catalytic amount of urea at 10 mol% (Sahiba et al. 2021; Sahiba and Agawal, 2020; Sahiba et al. 2020). The resulting compound was characterized by infrared (IR) spectroscopy, ^1^H and ^13^C nuclear magnetic resonance (NMR) spectroscopy and mass spectrometry (MS) (Figure 1).


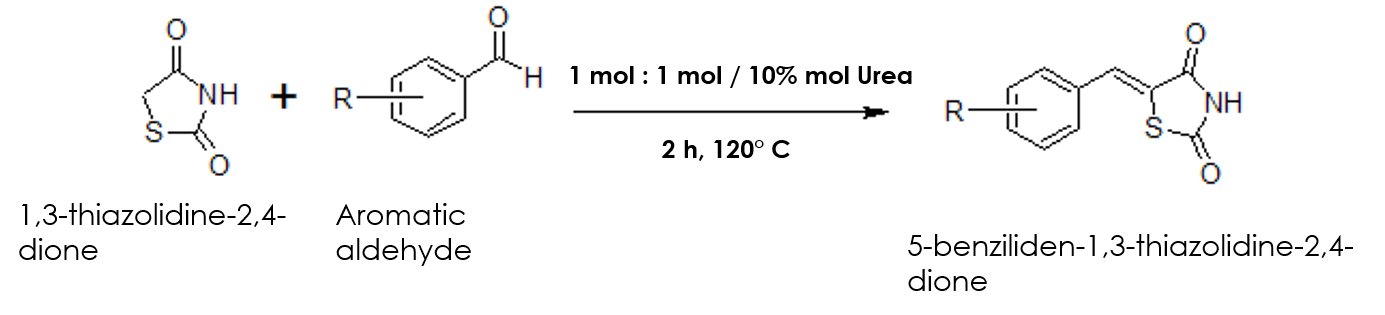


**Figure 1.** Synthesis by means of a Knoevenagel condensation.

- 1. Docking studies

The docking simulations reported by Alemán *et. al*. showed good theoretical affinity of C4 for the PPARγ receptor. Docking simulations were herein carried out on C4, C40 and C81. In brief, the structure of C4 was drawn with ChemDraw Ultra v10.0 (Cambridge Soft Corporation, USA), copied to Chem3D Ultra v10.0 to create a 3D model, and subjected to energy minimization on Gaussian 09 and GaussView v5 (Gaussian Inc, USA). The protein selected for molecular docking was the “A” monomer of entry 2PRG in the Protein Data Bank (PDB). Before docking the proposed compounds, the docking protocols of AutoDock v4.0 and AutoGrid v4.0 were validated by simulating the binding mode of a well-known crystallographic ligand (rosiglitazone). The results were visualized with Pymol 1.0 and VMD v1.8.7.

- 1. *Acute toxicity test of compound 4 (C4)*

Acute oral toxicity was evaluated according to protocol 425 of the OECD guideline. Healthy female albino Wistar rats, nulliparous and not pregnant, were randomly divided into groups to be given a range of doses. The animals were housed individually (otherwise kept under the previously described conditions, subsection 2.3) and acclimated to the lab conditions for 8 days prior to dosing. They were fasted overnight prior to the initial dose.

To determine toxicity by the up and down procedure, the compound was administered orally via orogastric cannula, employing doses increasing by a 0.5 progression factor (175, 350, 700, 1400 and 2000 mg/kg). Doses were selected based on the original protocol (annex 2, paragraph 2), which states that in the absence of an available estimate of the lethality of a given substance, dosing should be initiated at 175 mg/kg. In most cases, this dose is sublethal and therefore serves to reduce the level of pain and suffering of the animals. Since animal tolerance to the chemical was not expected to be highly variable, the dose progression factor was the default 0.5 on a log dose scale (3.2 factor). After administration of the compound, the behavior of the animals was monitored to detect any possible irregularities, especially neurological behavioral disturbances. The surveillance was carried out continuously for 5 h, frequently for another 5 h, and then occasionally for 24 or 48 h. To register survival, the animals were kept under observation for 14 days. The median lethal dose (LD_50_) was established with the Reed Muench formula.

1. Results
   1. *Docking studies*

One hundred and thirty derivatives were previously designed and subjected to docking simulations. The derivatives had the polar head of TZD and an aromatic mono and di-substituted body/tail portion.

Among the 117 crystals encoded for PPARγ and available in the PDB, entry 2PRG is the most complete, displaying the receptor co-crystallized with rosiglitazone as the ligand and SRC-1 as a coactivator. This murine PPARγ protein is homologous to human PPARγ, having 95% identity at the amino acid level.

Like rosiglitazone, the polar head of the test compounds interacted with His323, His449, Tyr473, Ser289 and Gln286. The partially lipophilic tail was inserted into the hydrophobic pocket of the ligand binding domain. In accordance with the physicochemical prediction, the proposed compound (C4) proved to be more hydrophilic and have a lower molecular weight than those employed as patterns (rosiglitazone, pioglitazone and troglitazone) (Tables 1 and 2).

**Table 1.** Docking scores for the standard compounds and the C4 derivative.

| **Compound** | **Molecular formula** | **Docking (kcal/mol)** | **No. of H bonds, residues and distances** |
| --- | --- | --- | --- |
| Rosiglitazone | C18H19N3O3S | -10.68 | 5 (His323/1.99 Å, His449/2.09 Å, Tyr473/3.01 Å, Ser289/1.72 Å, Gln286/1.86 Å) |
| Pioglitazone | C19H20N2O3S | -11.03 | 5 (His323/1.92 Å, His449/2.00 Å, Tyr473/3.07 Å, Ser289/1.70 Å, Gln286/1.88 Å) |
| Troglitazone | C24H27NO5S | -11.67 | 5 (His323/1.80 Å, His449/1.83 Å, Tyr473/3.35 Å, Ser289/1.88 Å, Gln286/2.24 Å) |
| C4 | C12H9NO2S | -8.06 | 5 (His323/1.85 Å, His449/1.85 Å, Tyr473/2.89 Å, Ser289/1.72 Å, Gln286/2.01 Å) |

**Table 2.** Physicochemical properties of the standard compounds and the C4 derivative.

| **Compound** | **Molecular formula** | **Molecular weight (g/mol)** | **Polar surface (Å)** | **H donors** | **H acceptors** | **log P** |
| --- | --- | --- | --- | --- | --- | --- |
| Rosiglitazone | C18H19N3O3S | 357.435 | 71.533 | 1 | 6 | 2.346 |
| Pioglitazone | C19H20N2O3S | 356.447 | 68.295 | 1 | 5 | 3.071 |
| Troglitazone | C24H27NO5S | 441.546 | 84.865 | 2 | 6 | 5.031 |
| C4 | C12H9NO2S | 231.276 | 49.93 | 2 | 4 | 2.240 |

*2.2 Synthesis of compound 4 (C4)*

The synthesis afforded a yellow dust with an Rf of 0.67 (hexane:ethyl acetate, 5:5), a final yield of 88.7%, and a melting point at 288 ± 2 °C. It showed good solubility in acetone, ethyl acetate, methanol, ethanol and dimethyl sulfoxide. The presence of the desired product, (5Z)-5-[(2E)-3-phenilprop-2-en-1-iliden]-1,3-thiazolidine-2,4-dione, was confirmed by IR, ^1^H and ^13^C NMR and MS. IR (cm^-1^): NH (3179.4), C=C-H (3028.2 y 2751), C=O (1729.04 y 1717.24) C=C (1677.5). ^1^H NMR (300 MHz, DMSO-d6): d/ppm12.4 (a, 1H, OH), 10.52 (s, 1H, NH), 7.99 (s, 1H, H6), 7.31 (d, 2J=1, 1H, H12), 7.28 (t, 3J=3, 1H, H10), 6.94 (d, 2J=2, 1H, H11), 6.92 (d, 2J=1, 1H, H9). ^13^C NMR (75.4 MHz, DMSO-d6): d/ppm 168.6 (C4), 168.0 (C2), 157.7 (C6), 132.7 (C11), 128.7 (C10), 127.4 (C12), 122.3 (C5), 120.3 (C9), 120.1 (C8), 116.5 (C7). MS 231.024.

*2.3 Acute oral toxicity test applied to C4*

The compound was administered at doses of 175, 350, 700, 1400 and 2000 mg/kg by orogastric cannula. The vehicle was ethanol (10%) and isotonic saline solution (90%) in a final volume of 1 mL. At all doses, the animals exhibited normal behavior and no visible physical changes. There were no significant findings in the post-mortem analysis. The LD_50_ of C4 was estimated to be over 77,090.34 mg/kg by the Reed Muench formula (Figure 2).

***
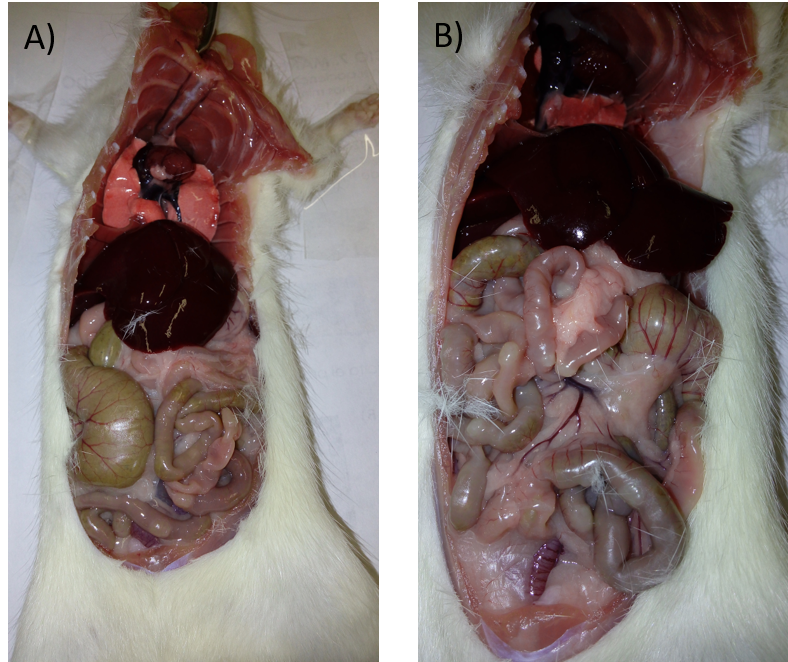
***

**Figure 2.** The gross necropsy of animals treated with C4 for the acute oral toxicity assay, performed as established by protocol 425 of the OECD guideline. A) 175 mg/kg and B) 2000 mg/kg of C4.

Based on the docking results, these three derivatives are able to interact with PPARγ. They were designed to have a lower molecular weight than the classic PPARγ agonists (rosiglitazone, troglitazone and pioglitazone) in order to diminish the adverse effects (e.g., hepatic toxicity) [Raikwar and Mishra 2020; Ajeet *et al*., 2018]. After the administration of C4, no toxic effect was found in any animal, which correlated with the high estimated value of its LD_50_. Hence, C4 was classified as category 5 on the scale of the Globally Harmonized System (GHS). C40 and C81 were previously reported to be category 5 and 4, respectively.

**References**

1. D. Alemán-González-Duhart, F. Tamay-Cach, J. Correa-Basurto, I. I. Padilla-Martínez, S. Álvarez-Almazán, and J. E. Mendieta-Wejebe, “In silico design, chemical synthesis and toxicological evaluation of 1,3-thiazolidine-2,4-dione derivatives as PPARγ agonists” *Regulatory Toxicology and Pharmacology*, vol. 86, pp. 25-32, 2017.
2. S. Agarwal, D. K. Agarwal, N. Gautam, K. Agarwal, and D. C. Gautam, “Synthesis and in vitro antimicrobial evaluation of benzothiazole incorporated thiazolidin-4-ones derivatives,” *Journal of the Korean Chemistry Society;* vol. 58, no. 1, pp. 33-38, 2014.
   1. Ajeet, A. Kumar, and A. K. Mishra, “Design, syntesis and pharmacological evaluation of sulfonamide derivatives screened against maximal electroshock seizure test.” *Molecular Biology*, vol. 7, pp. 206.
3. N. Raiwar and A. Mishra, “Prediction analysis of pharmacokinetic, toxicological and druglikeliness parameters of several oral hypoglycemic agents of sulfonyl ureas second generation using in silico metods.” *International Journal of Current Research in Medicines & Medical Science*, vol. 3, no. 1, pp. 4-10.
4. N. Sahiba, A. Sethiya, J. Soni, D. K. Agarwal, S. Agarwal, “Saturated five-membered thiazolidines and their derivatives: from synthesis to biological applications,” *Topics in Current Medicine*, vol. 378, no. 2, pp. 34, 2020.
5. N. Sahiba and S. Agarwal, “Recent advances in the synthesis of perimidines and their applications.” *Topics in Current Chemistry*, vol. 378, no. 44, pp. 1-47, 2020.
6. N. Sahiba A. Sethiya, J. Soni, and S. Agarwal, “Acridine-1,8-diones: synthesis and biological applications.” *Chemistry Select*, vol. 6, pp. 2210-2251, 2021.
